# User-Independent EMG Gesture Recognition Method Based on Adaptive Learning

**DOI:** 10.3389/fnins.2022.847180

**Published:** 2022-03-31

**Authors:** Nan Zheng, Yurong Li, Wenxuan Zhang, Min Du

**Affiliations:** ^1^College of Electrical Engineering and Automation, Fuzhou University, Fuzhou, China; ^2^Fujian Provincial Key Laboratory of Medical Instrument and Pharmaceutical Technology, Fuzhou, China; ^3^Fujian Provincial Key Laboratory of Eco-Industrial Green Technology, Wuyishan University, Wuyishan, China

**Keywords:** muscle synergy, user-independent, adaptive learning, surface electromyogram, pattern recognition

## Abstract

In a gesture recognition system based on surface electromyogram (sEMG) signals, the recognition model established by existing users cannot directly generalize to the across-user scenarios due to the individual variability of sEMG signals. In this article, we propose an adaptive learning method to handle the problem. The muscle synergy is chosen as the feature vector because it can well-characterize the neural origin of movement. The initial train set is composed of representative samples extracted from the synergy matrix of the existing user. When the new users use the system, the label is obtained by the adaptive K nearest neighbor algorithm (KNN). The recognition process does not require the pre-experiment for new users due to the introduction of adaptive learning strategy, namely, the qualified data and the label of new user data evaluated by a risk evaluator are used to update the train set and KNN weights, so as to adapt to the new users. We have tested the algorithm in DB1 and DB5 of Ninapro databases. The average recognition accuracy is 68.04, 73.35, and 83.05% for different types of gestures, respectively, achieving the effects of the user-dependent method. Our study can avoid the re-training steps and the recognition performance will improve with the increased frequency of uses, which will further facilitate the widespread implementation of sEMG control systems using pattern recognition techniques.

## 1. Introduction

Surface electromyogram (sEMG) signals contain information on muscular contractions, which is safe, non-invasive, and easy to be implemented. Hence, it has been widely utilized in human-computer interaction (HCI) based on gesture recognition (Ding et al., 2016a; Resnik et al., 2018; Ning et al., 2019). Compared with the image-based gesture recognition system, the sEMG-based gesture recognition system provides better portability and real-time performance. The HCI system based on sEMG signals consists of two main processes: first, a classification model is trained using a train set. Then, the classification model is used for gesture recognition and the result is used as the control input for HCI (Ding et al., 2016b). sEMG recognition method has been well studied during past decades, which can achieve more than 90% recognition accuracy for daily gestures (Tavakoli et al., 2018; Wahid et al., 2018; Jiang et al., 2020; Yang et al., 2021). However, due to the different muscle geometry, skin impedance, fat content, and maximal voluntary contraction of different users, sEMG signals can vary greatly when different users performed the same gestures (Jiang et al., 2012; Khushaba, 2014). Therefore, the sEMG model, trained with the sEMG data acquired from a specific user, can not perform well when applied directly to other users, which hinders the practical application of the HCI system based on the sEMG signals immensely.

Over the past few years, researchers have proposed two methods to deal with the aforementioned problems: the first method is to update the general model with a small amount of new user data. Khushaba (2014) applied canonical correlation analysis (CCA) in multi-user gesture recognition. Only a trial of the test user (for each movement) was used to map the train set and test set to a high correlation space. Xue et al. (2021) proposed a new framework CCA-OT that combined CCA and optimal transport (OT), which could further reduce the discrepancies in data distribution between the transformed feature matrix from the train set and the test set. Kim et al. (2020) introduced a user-independent gesture recognition method based on a muscle source activation model, which could only calibrate the model with a small subset of motions. In our previous study, a user-independent gesture recognition method, combined muscle synergy and the least square method (LSM) (Zheng et al., 2021), was proposed. The method combined a trial of the test data and LSM to obtain a transformation matrix, which would transform the train set. Although the above methods have an excellent performance in cross-user scenarios, the classifier still needs to be calibrated with some data from a new user, which will increase the use burden of new users. To improve the experience of a new user, some scholars shed the pre-experiment step and propose using deep learning models to mine user-independent features. Wei et al. (2019) proposed a multi-view convolutional neural network framework, by which an 82% recognition accuracy was achieved in NinaPro data sets. Chen et al. (2020) proposed a convolutional neural network based feature extraction approach (CNNFeat) and compared it with 25 traditional features. The results showed that CNNFeat outperforms all the tested traditional features in the inter-subject test. Recently, the idea of adaptive learning has been widely used in various fields (Luo et al., 2020; Chen et al., 2021; Wang et al., 2022). In gesture recognition, Li et al. (2019) applied incremental learning based on the Support Vector Machine (SVM) to solve the problem of individual variability and time variation of sEMG signals. The results showed that the incremental learning method could learn new knowledge from new users and significantly improve classification accuracy, however, the heavy training burden remains unsolved.

To sum up, the existing studies may have the following shortcomings:

i. The pre-experiment is required from new users, which will reduce the user experience. In practical application, balancing the relationship between the cost of pre-experiment acquisition and user experience is necessary.
ii. The large amounts of training data and complex computation are inevitable, which increase data acquisition costs and cause time-consuming.
iii. The time-varying nature of sEMG signals remains unsolved (Zhai et al., 2017), so the recognition accuracy will decrease with the onset of muscle fatigue.

In this study, a novel adaptive learning method is proposed to deal with multiple user problems. Inspired by incremental learning, we decide to update the train set with new user data evaluated by a risk evaluator. Considering that the KNN algorithm does not need a training process and can directly classify the query based on the information provided by the train set, the KNN algorithm is chosen as the basis of the classifier. First, the muscle synergy is extracted by Nonnegative Matrix Factorization (NMF) as a feature vector, and representative samples are extracted from the synergy matrix of the existing user using the k-means clustering algorithm. In this way, the amount of data can be reduced greatly. Then, the label of the test data is obtained by a designed KNN classifier that can adaptively adjust the *K*-values and weights. Finally, to overcome the individual differences of sEMG, the qualified data and their labels evaluated by the risk evaluator are used to update the weights and train set.

Different from related studies, the main innovations and contributions of this study lie in:

i. To avoid the pre-experiment process and to improve the experience of new users, qualified test data and the label are used to update the train set and KNN weight.
ii. To reduce the amount of data and the complexity of calculations, we extract muscle synergy as robust features and extract representative samples from the existing synergy matrix as the basis of classification.
iii. Updating the train set can learn the latest knowledge from new users, so it can adapt to the time-varying nature of sEMG signals, and the recognition accuracy will gradually increase with the frequency of uses, eventually achieving the level of the user-dependent model.

The remaining sections are arranged as follows. In Section 2, we describe the data set and processing procedures. In Section 3, we introduce the representative samples, adaptive KNN and risk evaluators, and describe how we apply the methods to our problem. Experimental results and discussions are demonstrated in Section 4. Finally, Section 5 concludes our study.

## 2. Data Set and Data Processing

### 2.1. Data Set

**Figure 1:**
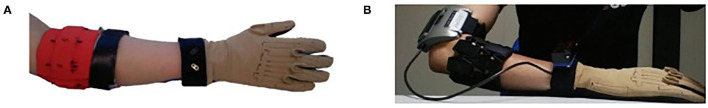
Acquisition setups and the placement: **(A)** DB1 OttoBock MyoBock 13E200, **(B)** DB5 Double Myo armband (Pizzolato et al., 2017).

The Database1 (DB1) and Database5 (DB5) of the NinaPro project (Atzori et al., 2014b) is used in this study. The DB1 and DB5 are constructed to apply to the research and development of the HCI system based on sEMG signals. The acquisition setups are OttoBock MyoBock 13E200 and Myo armband, respectively, which are placed in the extensor and flexor muscles of the fingers below the forearm elbow. The placement of electrodes is shown in Figure 1 and the detailed information of the database is shown in Table 1.

**Table 1:** Database information.

	**DB1**	**DB5**
Sample frequency	100 Hz	200 Hz
Number of channel	10	16
Number of persons	27	10
Number of repetitions	10	6
Movement	12 basic movements of the fingers	
	8 isometric,isotonic hand configurations	
	9 basic movements of the wrist	
	23 grasping and functional movements	

**Figure 2:**
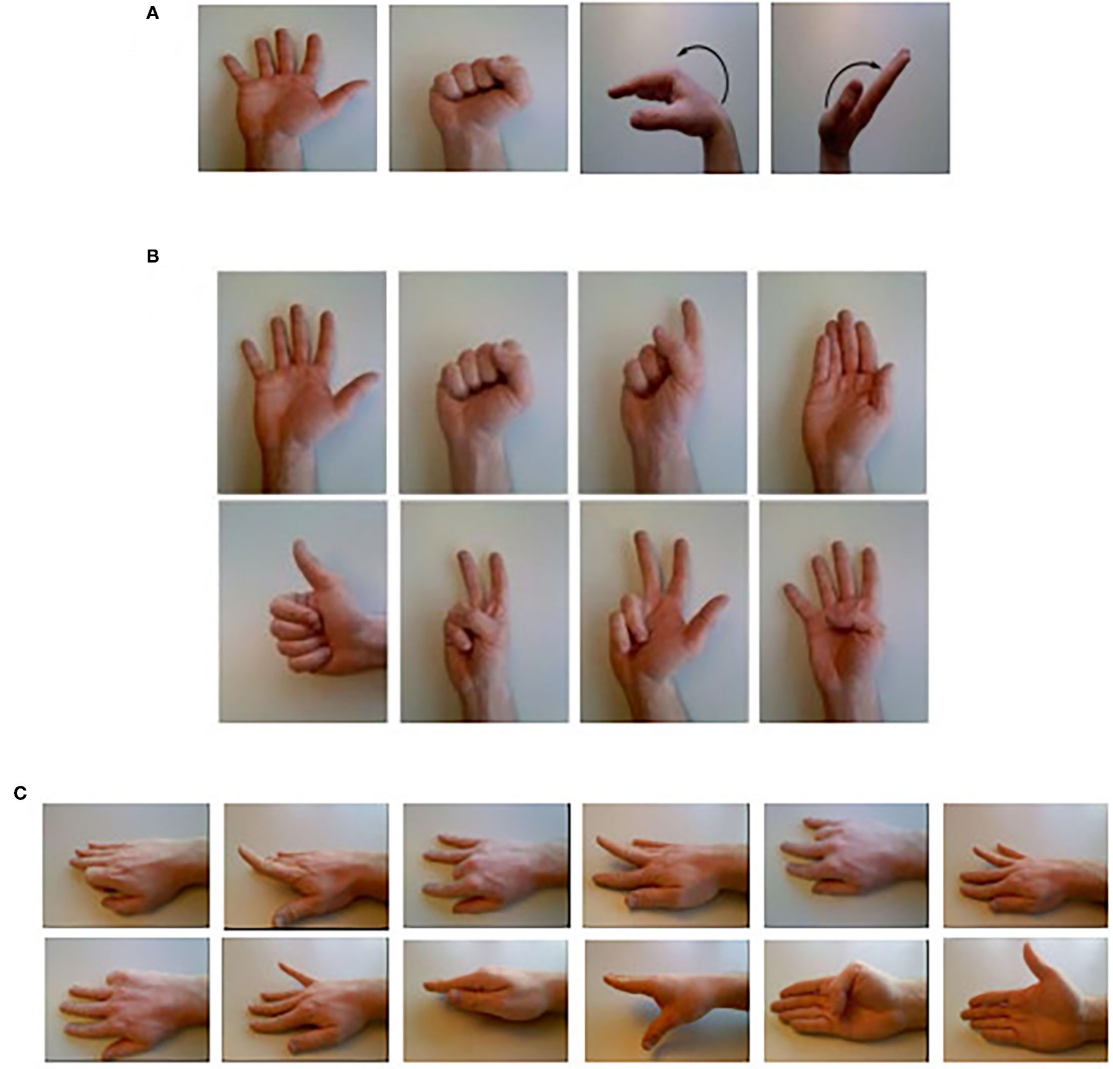
Twenty-two daily gesture: **(A)** four basic movements of the wrist and hand (Group one), **(B)** eight isometric, isotonic hand configurations (Group two), and **(C)** 12 basic movements of the fingers (Group three) (Atzori et al., 2014b).

From the 52 movements in the NinaPro database, we choose 22 daily gestures and divide them into three groups. Figure 2 graphically shows the selected gestures.

To compare with existing research using the same database, the experiments of group one are conducted with the DB5, while group two and group three are conducted with the DB1.

### 2.2. Data Preprocessing

The data preprocessing includes full-wave rectification, a three-order Butterworth low-pass filter with a cut-off frequency of 1 Hz, and active segment extraction. The envelope obtained by low-pass filtering is used to acquire active segment data. Taking DB5 as an example, a sliding window with a size of 200 ms (40 samples) was selected for the next steps, denoted as E(t). The threshold value is 0.015 times the peak value in the sliding window. When 35 samples in E(t) are all greater than the threshold value, the window is determined as an active window; otherwise, it is a resting window. Multiple active windows between two resting windows constitute an active segment. To improve the reliability of data, the active segments with apparent differences in repeated movements are discarded.

### 2.3. Muscle Synergy

Muscle synergy theory explains well how the nervous system recruit muscles to produce action. Combined with the analysis of muscle synergy in the previous study (Zheng et al., 2021), muscle synergy is selected as the feature of the sEMG signal.

To ensure the real-time performance of online recognition, a sliding window with a size of 200 ms overlapped by 50 ms is chosen for online recognition (Jaramillo-Yánez et al., 2020). For each sliding window, the sEMG signal can be decomposed into two matrices by NMF (Teng et al., 2021):


(1) $$\begin{array}{l} V=W\times H \end{array}$$

Where ***V*** is the *m*×*n* initial envelope signal matrix (*m* is the number of muscles, *n* is the length of the muscle activation pattern), ***W*** is the *m*×*r* synergy matrix (*r* is the number of synergies), and ***H*** is the *r*×*n* coefficient matrix.

***W*** and ***H*** can be solved by (2) and (3):


(2) $$\begin{array}{l} W\left(io\right)=W\left(io\right)\frac{\left[VH^{T}\right]\left(io\right)}{\left[WHH^{T}\right]\left(io\right)} \end{array}$$

(3) $$\begin{array}{l} H\left(oj\right)=H\left(oj\right)\frac{\left[W^{T}V\right]\left(oj\right)}{\left[W^{T}WH\right]\left(oj\right)} \end{array}$$

Where *i* = 1, 2, 3⋯*m*, *o* = 1, 2, 3⋯*r*, and *j* = 1, 2, 3⋯*n*. According to the references (Zheng et al., 2021), the optimal number of muscle synergies is 1, so *r* = 1.

Finally, to unify the dimensions of the features, all sEMG features are normalized as shown in Formula (4):


(4) $$\begin{array}{l} X_{norm}=\frac{X-X_{\min}}{X_{\max}-X_{\min}} \end{array}$$

Where ***X*****_*norm*_** represents the normalized sEMG features, ***X*** denotes the sEMG features, ***X*****_*max*_** and ***X*****_*min*_**, respectively, are the maximum value and minimum value of ***X***.

## 3. Methods

### 3.1. The Proposed Adaptive Method for Gesture Recognition

**Figure 3:**
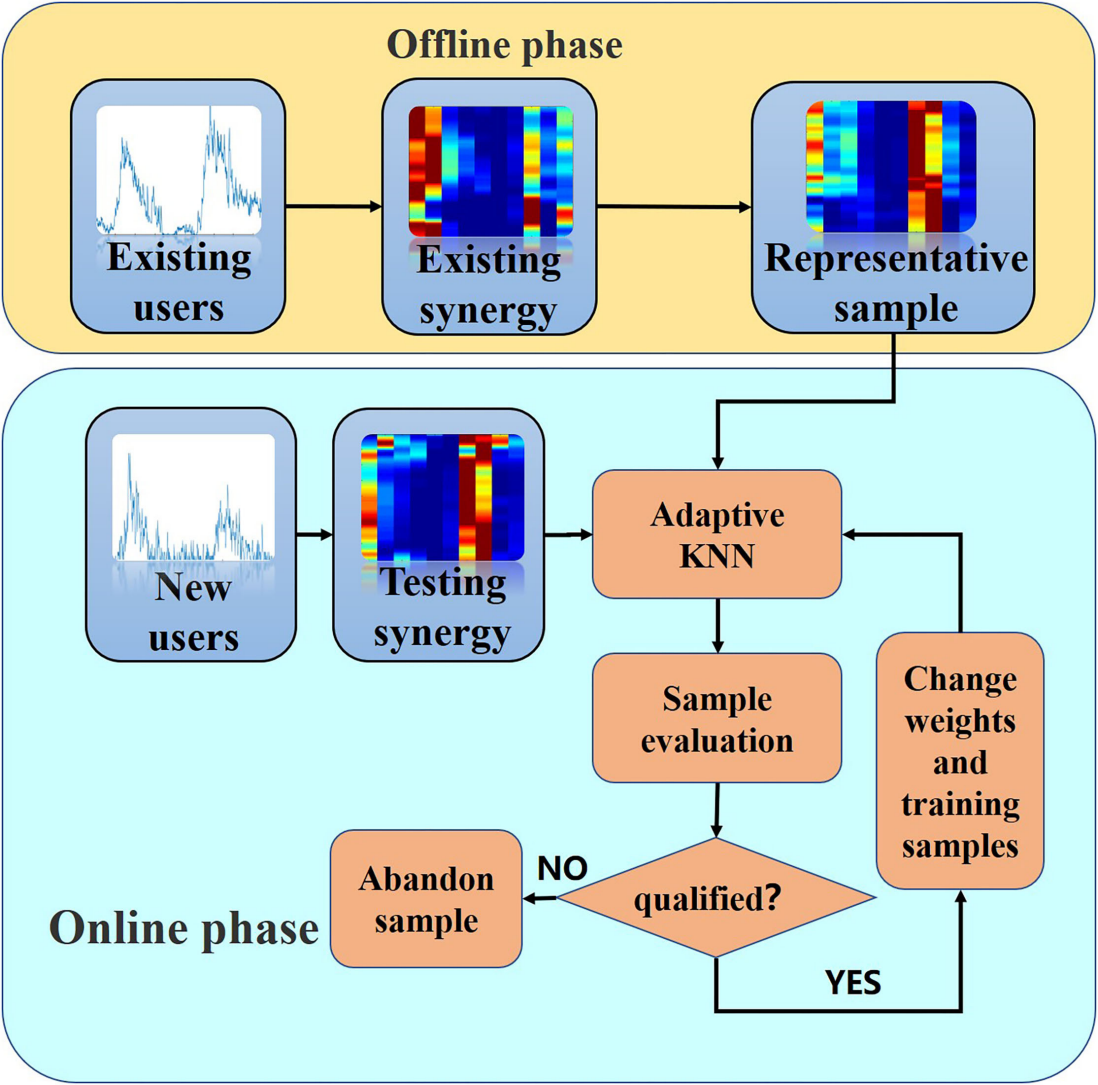
The framework for user-independent gesture recognition.

The overall framework of adapting learning for gesture recognition is shown in Figure 3.

It can be seen from Figure 3, our proposed framework consists of two parts: offline processing and online recognition. In the offline phase, we first extract muscle synergy (denoted as existing synergy) from existing user data by NMF algorithm. The existing user data is removed by noise signal by data processing in advance. Then, the clustering center of each action is extracted using the clustering method, and the nearest sample of the existing synergy to the clustering center is extracted as the representative sample, which is the basis of the next steps.

In the online stage, the test data, after the same data processing as in the offline phase, obtain the labels by the KNN classifier. Then, the test data is evaluated by a risk evaluator, and the qualified one and its labels are used to update the weights and replace the remote samples of the train set, whereas those samples that do not meet the requirements are discarded.

### 3.2. The Method of Extracting Representative Sample Based on K-Means Algorithm

K-means algorithm is an unsupervised clustering method (Yu et al., 2020), which classifies the class by the distance between sample and centroid. In this study, the K-means algorithm is used to extract the template of each gesture, and then the representative samples of each gesture can be obtained. Assuming that the test data is *x*, the cluster is *C*_*i*_ ( *i* = 1, 2, 3⋯*N*, *N* is the number of gesture categories), and the centroid of the cluster *C*_*i*_ is μ_*i*_, the optimization objective is to minimize the error ***E***:


(5) $$\begin{array}{l} E=\overset{N}{\underset{i=1}{\sum }}\underset{x\in C_{i}}{\sum }||x-\mu_{i}|{|}_{2}^{2} \end{array}$$

(6) $$\begin{array}{l} \mu_{i}\text{=}\frac{1}{\left|C_{i}\right|}\underset{x\in C_{i}}{\sum }x \end{array}$$

To solve the Formula (5), the vector mean of each gesture is chosen as the initial centroid. We set the upper limit of iterating as 100, and the procedure terminates when several iterations are reached or the difference between the centroid of the last generation and the centroid of the previous generation is <0.02. In the process of iterations, the centroid of each generation was recorded.

The final centroid of each gesture is chosen as the template, and we select the 30 samples closest to the template as a representative sample for each gesture of each user. Taking DB5 as an example, the dimension of the initial train set is 12,312 ×16, and the dimension of the representative sample is 1,080 × 16, which greatly reduces the amount of the train set.

### 3.3. Adaptive KNN Algorithm

Traditional incremental learning methods discard samples after learning some characteristics of samples, which will result in information loss. To make full use of the new user data, this article proposes to update the train set directly by using the new user data. As a typical case-based algorithm, the KNN algorithm only needs to compare the distance between the test set and the train set to get the label during classification. Therefore, there is no need to train the model again after updating the train set, which significantly improves the efficiency of the algorithm.

The traditional KNN algorithm searches for the points closest to the test sample in the train set and counts the categories of the nearest points. The category with the most significant number is the category of the test sample. The method is simple and effective but it also has some disadvantages: First, the *K*-value of the traditional KNN algorithm is fixed, which is not suitable for samples with uneven distribution (Mullick et al., 2018; Pan et al., 2020). Second, the KNN algorithm has the same weight on each neighbor point without considering the contribution of different samples to the classification (Gou et al., 2014).

To address the problems of the traditional KNN algorithm, this article proposes an improved method that automatically selects the appropriate *K*-values according to the distribution of samples and adjusts the weights according to the contribution of samples to the classification.

**Figure 4:**
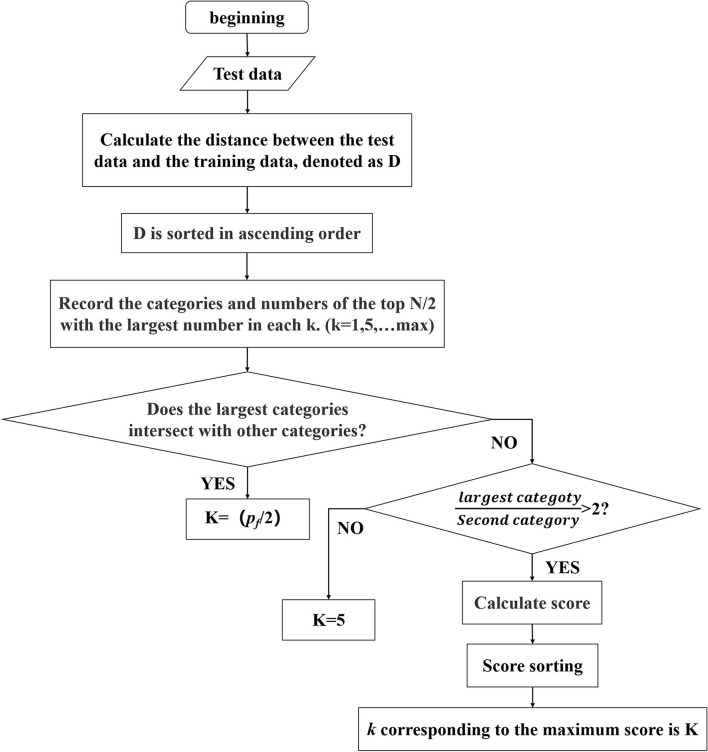
The process of adapting *K*-value.

The process of the adaptive *K*-value is shown in Figure 4. It mainly has three stages. First, the distances between the test sample *x* and the train set ***Y*** = [*y*_1_, *y*_2_, ...*y*_*n*_] are calculated and sorted in ascending order:


(7) $$\begin{array}{l} D_{i}=\sqrt{{\left(x-y_{i}\right)}^{2}} \end{array}$$

where *i* = 1, 2, 3⋯*q* , *q* is the number of samples in the train set.

Then, the *k* value of each time is a multiple of 5, and the top *N*/2 categories with the largest number in *k* are recorded each time. *N* is the number of gesture categories.

Finally, we judge whether the largest category has remained the same. If not, it means that the distribution of different gestures overlaps together. In this condition, a larger *K*-value indicates that more weights have to be updated, which will bring great risks. The first intersection between the largest category and other categories is recorded, denoted as *p*_*f*_. So, K can be obtained as follow:


(8) $$\begin{array}{l} \text{K}=\frac{p_{f}}{2} \end{array}$$

If there are no intersections between the largest category and other categories, the ratio of the maximum number of categories to the second category is calculated. If the ratio is <2, we set K = 5; If it is >2, we calculate the score of each k according to Formula (9) and sort the score, and *k* corresponding to the maximum score is K.


(9) $$\begin{array}{r} \text{score}=\frac{v}{\max\left(v\right)}+\frac{den}{\max\left(den\right)} \end{array}$$

(10) $$\begin{array}{l} v=\frac{s}{k},\;den=\frac{k}{\pi d^{2}} \end{array}$$

where *s* is the number of the largest category and *d* is the longest distance in the largest category sample.

In Formula (10), *v* denotes purity, and *den* denotes density. It is normalized to avoid an order of magnitude too large for one of the parameters.

The approach to adaptive weights can be introduced through three steps. First, the initial weight of 1 is assigned to each sample. Then, the label of the test samples is obtained by the adaptive *K*-value KNN algorithm. Finally, the label of the qualified samples (refer to Section 3.4) is used to update the weight.

The weight of the sample will increase if it is chosen by the classification steps and its label is the same as the label of qualified samples. To avoid the weight of one sample being too large to cover other samples, it is considered to add amplitude limiting. The increased weight is as follows:


(11) $$\begin{array}{l} q_{new1}=\frac{3}{1+e^{-q_{old}}} \end{array}$$

On the contrary,we reduce the weight of our sample if these samples are selected by the classification steps but its label is different from that of qualified ones. The weight should not be negative, so the weight is reduced proportionally.


(12) $$\begin{array}{l} q_{new2}=\frac{q_{old}}{4} \end{array}$$

**Figure 5:**
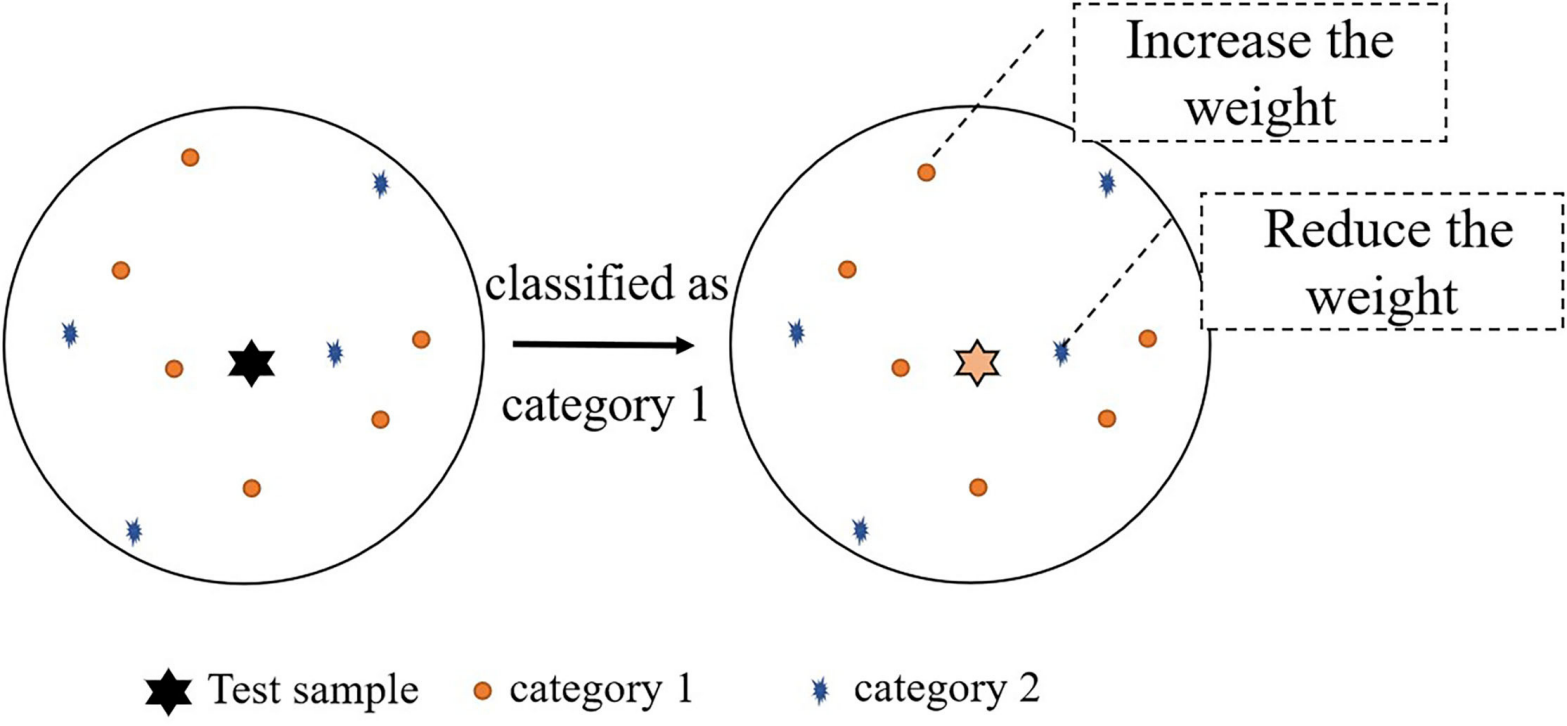
Schematic diagrams of updating the weight.

In the case of K = 10, the diagram of updating the weight is shown in Figure 5. As can be seen from the graph, the number of Category 1 is greater than the number of Category 2, so the test sample is classified as Category 1. In the circle of K = 10, the weight of Category 1 is updated by *q*_*new*1_, and the weight of Category 2 is updated by *q*_*new*2_.

### 3.4. Risk Assessors

In this study, we decide to update the train set with new user data, so the new user data should be evaluated by the risk evaluator. Only qualified samples can be used to update the train set. The process of evaluating is as follows: First, the partial train set corresponding to the label of the test sample is extracted, and the farthest point between the partial train set and the template is calculated and denoted as *p*_*s*_.

Then, let test sample *x* and *p*_*s*_ be *A* and calculate the risk using the following Formula (13).


(13) $$\begin{array}{l} risk=\frac{1}{\frac{\overset{m}{\underset{i}{\sum }}A_{i}\mu_{1i}}{\sqrt{\overset{m}{\underset{i=1}{\sum }}A_{i}^{2}}\sqrt{\overset{m}{\underset{i=1}{\sum }}\mu_{1i}^{2}}}\text{+}\overset{4}{\underset{j=2}{\sum }}\sqrt{\overset{m}{\underset{i}{\sum }}{\left(A_{i}-\mu_{ji}\right)}^{2}}} \end{array}$$

where μ_1_ represents the templates of the categories corresponding to the labels, μ_*j*_(*j* = 2, 3⋯*N*) represents the templates of the other categories. Additionally, the number of muscles *m* is the characteristic length.

In the above formula, the first term of the denominator represents the degree of similarity intra the class, and the second term represents the degree of difference inter the classes.

In the case that the risk value of the test sample is less than the farthest point, we think that the test sample has better inter class distance and intra class distance. Therefore, the farthest point is replaced by the test sample to update the train set, making the train set constantly learn new user features. Meanwhile, the weight of the qualified sample is initialized.

### 3.5. Evaluation

The classification performance of the algorithm in DB1 and DB5 is evaluated by the classification accuracy, i.e., the number of correctly classified samples divided by the total number of samples.

The data processing and classification using MATLAB R2018A, and a one-way Analysis of Variance (ANOVA) are used to analyze the experimental results. The *p* < 0.05 shows a significant difference.

### 3.6. Cross-Validation

To verify whether the recognition accuracy will improve with the increased frequency of uses, each time a group of repetitive actions of new users is randomly selected for testing until all the groups of repetitions are tested, which is recorded as a cycle. To make the results more objective, 10 cycles are conducted, and the average of 10 cycles is the final recognition rate of the new user.

To avoid the influence of random selection on the objectivity of the results, we use *M*-fold cross-validation, *M* is the number of users. Each time *M*-1 user is selected as an existing user, and the remaining user is considered as a new user. This process is repeated *M* times until each user is treated as a new user. Finally, the average value of the result is the accuracy of gesture recognition.

## 4. Results and Discussion

### 4.1. The Results of Extracting the Envelope and Active Segment

**Figure 6:**
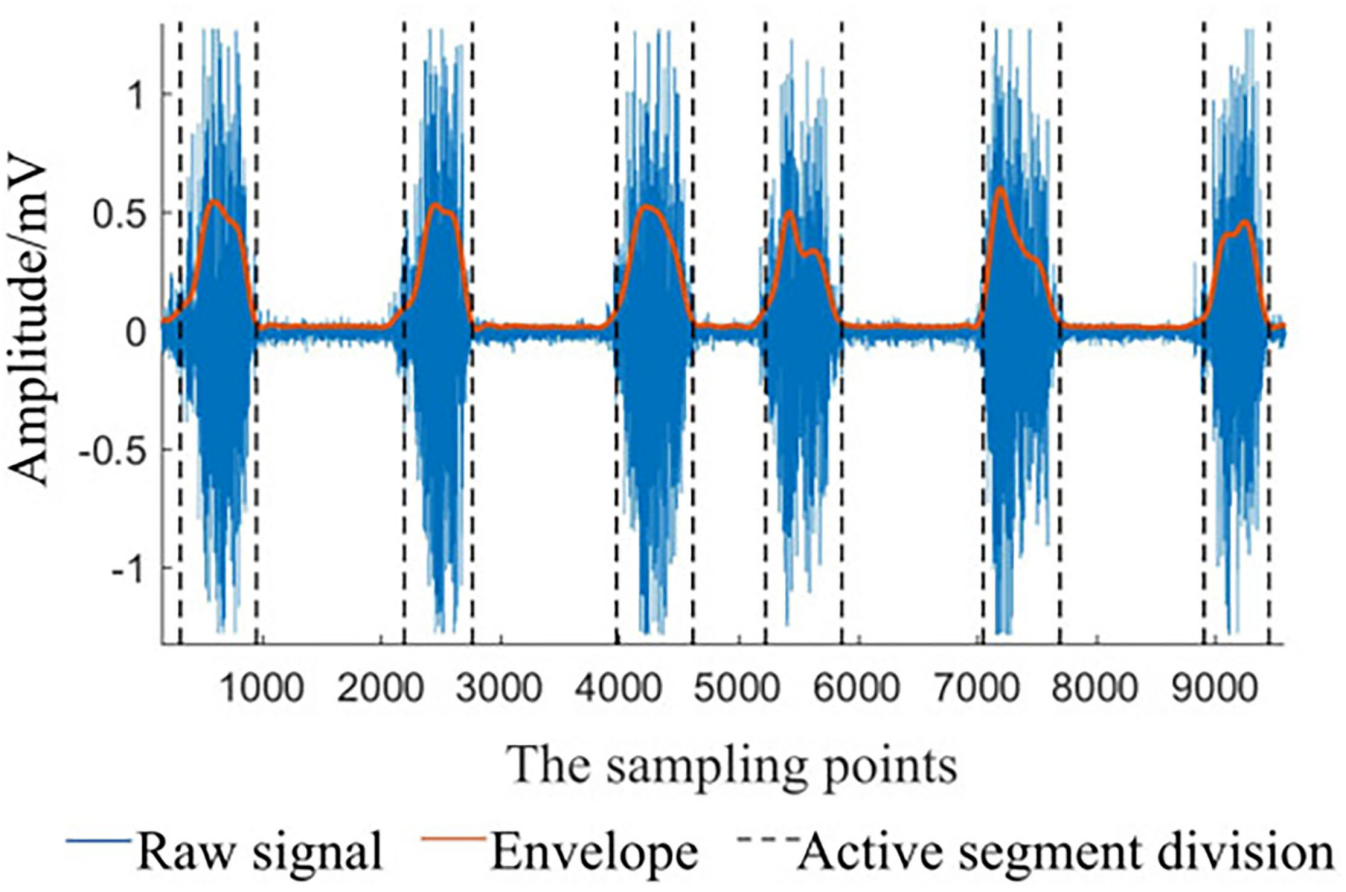
Envelope and activity segment.

Taking the hand closed motion of DB5 as an example, according to the method in Section 2, the result of signal preprocessing is shown in Figure 6. It can be seen that the algorithm can extract the active segment well and make the signal more smoother, which is conducive to subsequent classification.

### 4.2. The Relationship Between Frequency of Use and Recognition Accuracy

**Figure 7:**
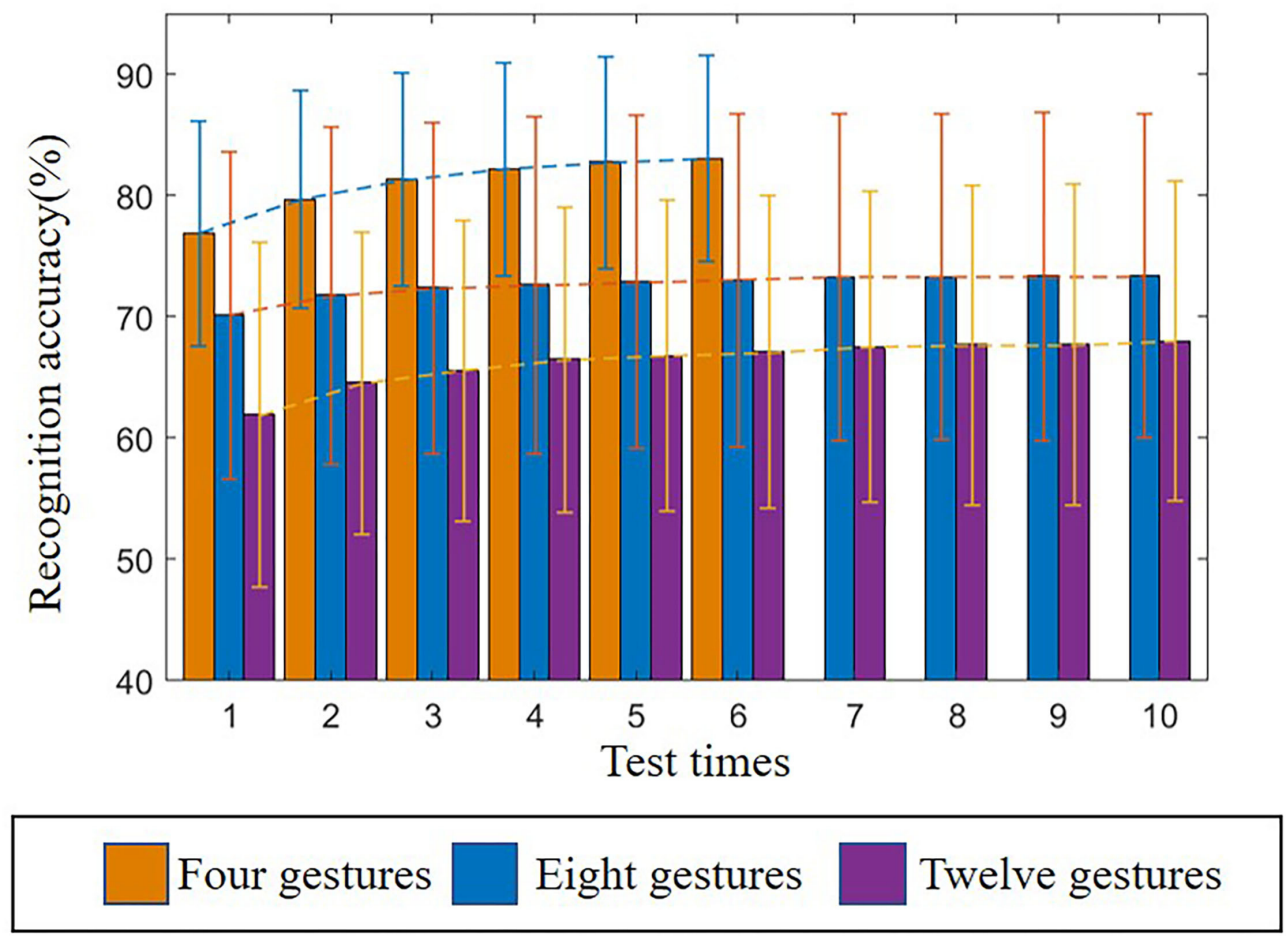
Relationship between average recognition accuracy and test times.

The adaptive learning proposed in this article is mainly reflected in replacing poor samples with qualified samples in the train set and updating weights. Since the classifier learns the characteristics of new users, the recognition performance should increase with the frequency of use. The average recognition accuracy of 10 users (DB5) and 27 users (DB1) improves with the increased frequency of use, as shown in Figure 7.

According to this histogram, with the increase of the number of tests, the average recognition accuracy of the three group's gestures increase steadily, which proves that the method of replacing the bad samples with the qualified samples can effectively improve the recognition accuracy, and it can adapt to the time-varying characteristics of sEMG during exercise. However, Figure 7 also reflects the slow growth rate of the accuracy. The possible reason is that the classifier has learned enough new user features, and the recognition accuracy is close to the user-dependent model. Therefore, the increase of data is of limited help to improve the recognition accuracy.

### 4.3. Changes of Channel Weights at Different Stages

**Figure 8:**
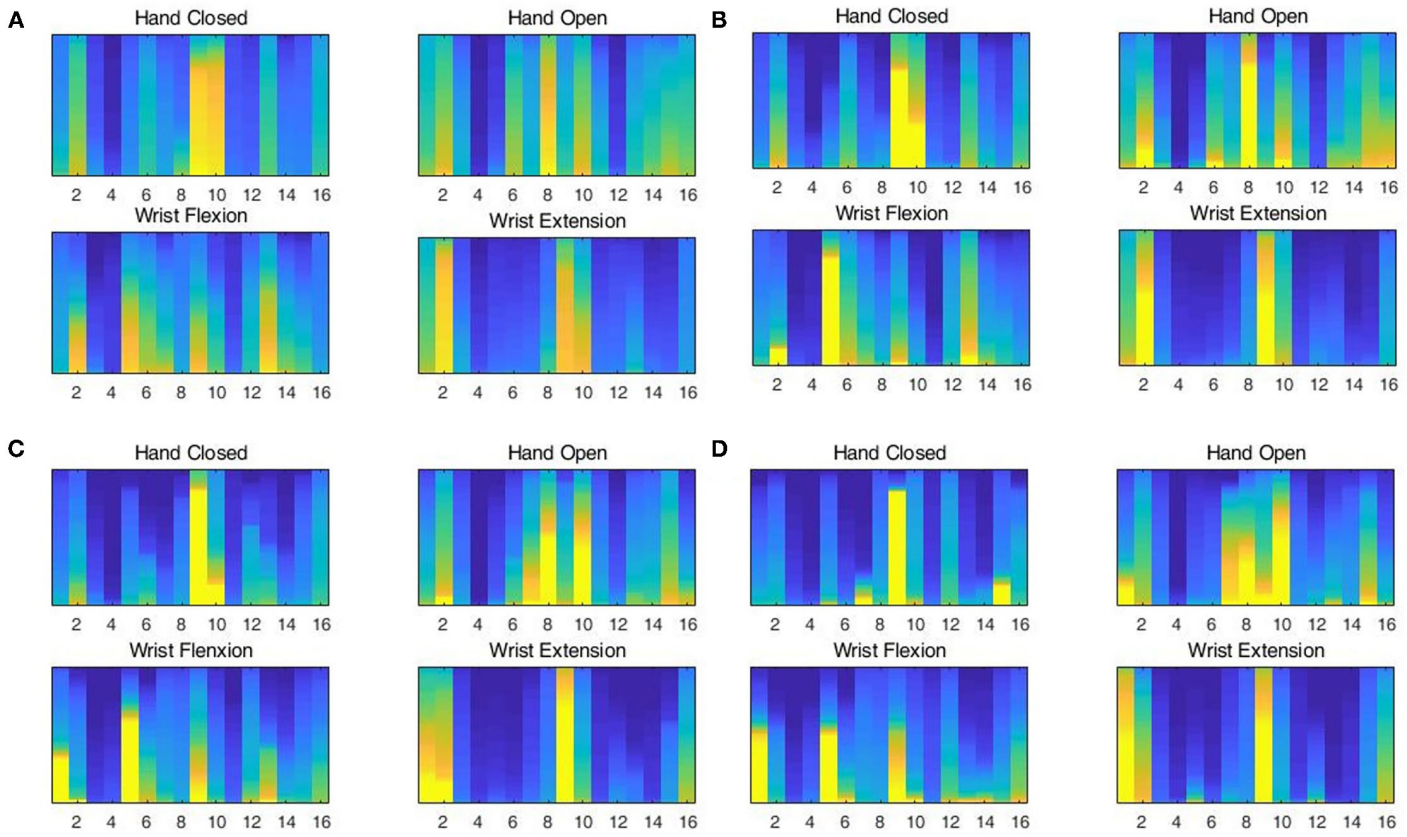
Changes in channel weights at different stages of **(A)** the existing samples, **(B)** the initial train set, **(C)** the updated train set, and **(D)** test samples.

If the user-independent gesture recognition method wants to reach a good recognition accuracy, the distribution of the train set must be similar to the test set. In this article, we use the weights of the channels to measure the similarity of the dataset with the new user data at different stages. The changes in weight are shown in Figure 8.

Figure 8 shows that different hand gestures will activate different channels. In Figure 8D, for test data, hand closed movement mainly activates channel 9, hand open movement mainly activates channels 8 and 10, wrist flexion movement mainly activates channels 1, 5, and 9, and wrist extension movement mainly activates channels 1 and 9. For the existing data without processing, as shown in Figure 8A, the activated channels are significantly different from the test sample. It can be seen from Figure 8B, the representative sample (denoted initial train set) discards remote samples from the existing synergy matrix, so the channel weights are more similar to the test set, but the initial train set does not learn the distribution of the test sample, so it still fails to meet the requirements. Test samples that pass the risk evaluator evaluation can be used to replace remote samples in the initial train set, so the updated train set begins to resemble the distribution of the test set. As can be seen from Figure 8C, hand closed movement mainly activates channel 9, hand closed movement mainly activates channels 8 and 10, wrist flexion movement mainly activates channels 1, 5, and 9, and wrist extension movement mainly activates 1, 2, and 9. Although there are differences in the degree of activation, the differences are at an acceptable level.

### 4.4. Gesture Recognition Results

#### 4.4.1. Comparison With Benchmark Algorithm

To verify the effectiveness, advantages, and performance of the proposed algorithm, three benchmark comparison schemes, including two user-independent schemes and one user-dependent scheme, are set up as follows:

Scheme A (user-independent): one user is selected at a time as a new user, and the remaining user is an existing user. Muscle synergy is extracted as the feature, and muscle synergy of all existing users is used to train the model, while muscle synergy of new users is used to test the model.

Scheme B (user-independent): one user is selected as the new user, and the remaining user is the existing user. Muscle synergy is taken as a feature. Representative samples of existing user data are extracted according to Section 3.2. The representative samples are used to train the model, and muscle synergy of new users is used to test the model.

Scheme C (user-dependent): for each user, muscle synergy is extracted as a feature, 70% of all synergy is used to train the model, and the remaining 30% is used to test the model.

Scheme D is the algorithm presented in this article.

**Figure 9:**
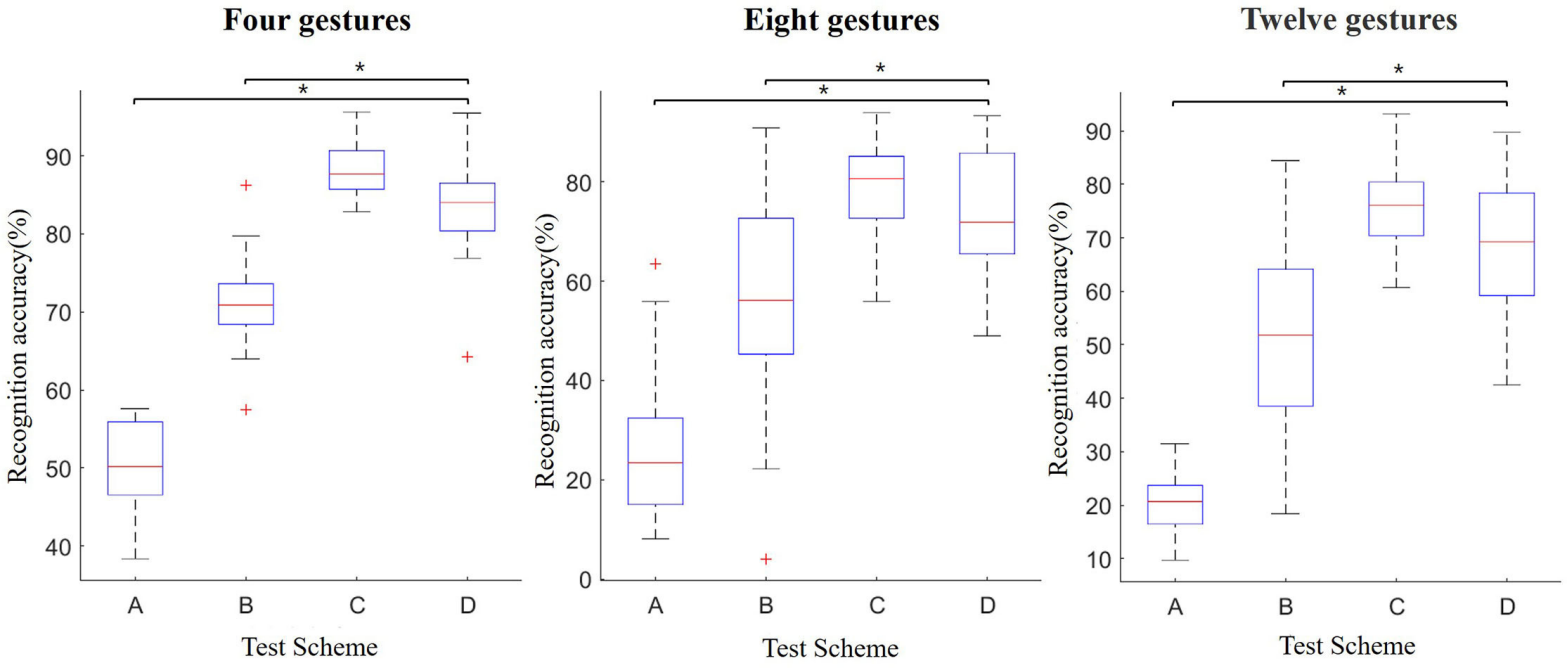
Comparison of significance among different schemes (*p < 0.05).

The above schemes are applied to three groups of gesture recognition experiments, and the result is shown in Figure 9.

According to Figure 9, in the three groups of gesture experiments, the recognition accuracy of the proposed method (scheme D) is 35% higher than scheme A. Meanwhile, there is a significant difference between scheme A and scheme D (*p* = 1.17e-08, *p* = 3.35e-17, *p* = 5.69e-23). The result indicates that the proposed method can significantly improve the user-independent recognition performance.

The averaged classification rates in three groups of gesture experiments are 61.31 and 74.67%, corresponding to scheme B and scheme D. Our method achieves significantly better results as validated by an ANOVA test with the achieved *p* < 0.05 against scheme B (*p* = 0.005, *p* = 0.001, and *p* = 0.0004). The comparison results show that based on representative samples in this article, updating the train set and weight can effectively improve the recognition accuracy.

Although the recognition accuracy is lower than scheme C, there is no significant difference between scheme C and scheme D (*p* = 0.09, *p* = 0.07, and *p* = 0.05). This shows that the performance of the proposed method can achieve the level of the user-dependent model, which also confirms the conjecture that we proposed in the analysis of Figure 7. From a methodological point of view, although using qualified samples to replace remote samples directly can improve the recognition accuracy, some samples will inevitably be updated with the wrong labels. This part of the sample results in the accuracy being lower than the user-dependent model.

#### 4.4.2. Comparison of Results With Other Research

The research results of user-independent gesture recognition using the same database as in this article are shown in Table 2.

**Table 2:** Comparison of results with other searches.

**Group**	**Data source**	**Method**	**Sample frequency**	**Recognition accuracy (%)**
Group one	DB5	Zheng et al., 2021	200	**83.25**
	DB5	Our	200	83.05
	DB1	Patricia et al., 2014	100	40.00
Group two	DB1	Du et al., 2017	100	67.40
	DB1	Padhy, 2020	100	**75.23**
	DB1	Our	100	73.35
	DB1	Patricia et al., 2014	100	25.00
Group three	DB1	Atzori et al., 2014a	100	55.00
	DB1	Ketykó et al., 2019	100	65.45
	DB1	Our	100	**68.04**

*The bold values represent the literature in the same group with the highest accuracy*.

Table 2 shows that the recognition accuracy of the proposed method is slightly lower than the references Padhy (2020) and Zheng et al. (2021) in Group one and Group two, but it is higher than the existing literature in Group three. Padhy (2020) proposed a tensor-based approach using multilinear singular value decomposition (MLSVD) for hand gesture recognition. This method achieved good recognition accuracy in user-independent gesture recognition, but it required larger computational resources because of the complex transformation relationship. Meanwhile, the sliding window length was 150 ms, and the average processing time of each channel was 199 ms. It demonstrated that the recognition time of a single gesture was over 300 ms, which did not meet the requirements of real-time for gesture recognition. Zheng et al. (2021) was preliminary work of our team. The combination of a trial (for each movement) for new users and LSM was adopted to update the train set. This method had great recognition accuracy and computation advantages compared with other studies. In this article, we extract representative samples to reduce the amount of data further. At the same time, we use adaptive learning to adapt to the feature distribution of new users, which can avoid the pre-experiment step for new users. We optimize preliminary work in two aspects, and the final recognition accuracy is only slightly lower than the preliminary work. Therefore, the optimization strategy proposed in this article is effective, and the biggest advantage of this method is that it completely avoids the pre-experiment process required in other studies.

## 5. Conclusion

In this article, an adaptive learning gesture recognition method is proposed to solve the user-independent problem. To simplify the calculation and eliminate abnormal samples, K-means clustering is first used to extract representative samples from existing synergy. Then, the label of the test data can be obtained by the adaptive KNN classifier. Finally, we evaluate the test sample by risk evaluator, and the qualified samples and their labels are used to update the weight and train set. Our method is analyzed by three comparisons in different directions, such as before-and-after processing comparison, comparison of various schemes, and comparison of different algorithms. All the results prove the effectiveness of the proposed method. The method proposed in this article not only improves the classification accuracy but also adapts to the time variability of the sEMG signal. In the practical application, the pre-experiment does not request new users, which is conducive to the promotion of sEMG signal-based HCI systems. Inspired by the literature (Zeng et al., 2020, 2021), the optimization of the risk evaluator, the template, and the neighborhood will be our next investigation to further enhance the user-independent sEMG classification results.

## Data Availability Statement

The original contributions presented in the study are included in the article/supplementary material, further inquiries can be directed to the corresponding author/s.

## Author Contributions

NZ and WZ wrote this manuscript. NZ, YL, and WZ conduced the conception of the study. YL and MD revised the manuscript critically for important intellectual content. All authors reviewed, revised, and finalized the manuscript. All authors contributed to the article and approved the submitted version.

## Funding

This study was supported in part by the Fujian Province Nature Science Foundation of China under Grant 2019J01544 and in part by the National Nature Science Foundation of China under Grant 61773124.

## Conflict of Interest

The authors declare that the research was conducted in the absence of any commercial or financial relationships that could be construed as a potential conflict of interest.

## Publisher's Note

All claims expressed in this article are solely those of the authors and do not necessarily represent those of their affiliated organizations, or those of the publisher, the editors and the reviewers. Any product that may be evaluated in this article, or claim that may be made by its manufacturer, is not guaranteed or endorsed by the publisher.
